# Stability Study of Fosfomycin in Elastomeric Pumps at 4 °C and 34 °C: Technical Bases for a Continuous Infusion Use for Outpatient Parenteral Antibiotic Therapy

**DOI:** 10.3390/pharmaceutics15092347

**Published:** 2023-09-19

**Authors:** Alessandra Manca, Alice Palermiti, Jacopo Mula, Jessica Cusato, Domenico Maiese, Marco Simiele, Amedeo De Nicolò, Antonio D’Avolio

**Affiliations:** 1 Laboratory of Clinical Pharmacology and Pharmacogenetics, Department of Medical Sciences, Amedeo di Savoia Hospital, University of Turin, Corso Svizzera 164, 10149 Turin, Italy; alessandra.manca@unito.it (A.M.); alice.palermiti@unito.it (A.P.); jessica.cusato@unito.it (J.C.); domaiese08@gmail.com (D.M.); amedeo.denicolo@unito.it (A.D.N.); antonio.davolio@unito.it (A.D.); 2 CoQua Lab s.r.l., Corso Svizzera 185 bis, 10149 Turin, Italy; marco.simiele@coqualab.it

**Keywords:** Fosfomycin, UHPLC, stability, OPAT, bridge therapy

## Abstract

Background: Fosfomycin acts against aerobic Gram−/+ bacteria by blocking the synthesis of peptidoglycan. Its use has been currently re-evaluated for intravenous administration for the treatment of systemic infections by multidrug-resistant bacteria. Concentration-/time-dependent activity has been suggested, with potential clinical advantages from prolonged or continuous infusion. Nevertheless, little is known about Fosfomycin stability in elastomeric pumps. The aim of the present work was stability investigation before administration at 4 °C and during administration at 34 °C. Methods: InfectoFos^®^ (InfectoPharm s.r.l., Milan, Italy) preparation for intravenous use in elastomeric pumps at 4 °C and 34 °C was analyzed following EMA guidelines for drug stability. Samples were analyzed with an ultra-high performance liquid chromatography coupled with tandem mass spectrometry method on a LX50^®^ UHPLC system equipped with a QSight 220^®^ (Perkin Elmer, Milan, Italy) tandem mass spectrometer. Results: Fosfomycin in elastomeric preparation is stable for at least 5 days at a storage temperature of 4 °C and 34 °C. Conclusions: The results suggest Fosfomycin eligibility for continuous infusion even in the context of outpatient parenteral antibiotic therapy. Therefore, this approach should be tested in clinical and pharmacokinetic studies, in order to evaluate the possible gains in the pharmacokinetic profile and the clinical effectiveness.

## 1. Introduction

It is known that Fosfomycin (FOS) is a natural antibiotic produced by *Streptomyces*, discovered for the first time in 1969 by a Spanish team from the Spanish Penicillin and Antibiotics Company (CEPA) [1,2].

At first, it was isolated by screening a strain database consisting of actinomyces, including *Streptomyces fradiae*, for detecting potential inhibiting activity in cell wall biosynthesis and formation of growing bacteria [3], but it was also identified in other microorganisms [4].

FOS is a highly polar phosphonic acid derivative (cis-1,2-epoxypropyl phosphonic acid) that represents its own class of antibiotics [5].

FOS is a molecule with a low molecular weight (138 g/mol) and with an acid profile. It shows negligible protein binding (ca. 0%), and it easily distributes to most of the tissues and to the interstitial fluid. Therefore, it can penetrate inflamed tissues and bones but also lungs, reaching good concentrations [6,7,8].

FOS was initially commercialized as calcium salt formulation: FOS calcium for oral administration and FOS disodium, a higher hydrophilic salt, for parenteral administration. Then, FOS tromethamine, which reports a higher bioavailability (30–40%), was marketed and has become the standard formulation for oral administration.

According to its chemical/physical characteristics, FOS shows a wide volume of distribution (approximately 0.3 L/kg) and is eliminated via glomerular filtration; for this reason, some potentially, clinically relevant variability can be observed in critically ill patients, who could experience major changes in membrane permeability and in renal function [5,9,10].

FOS acts against aerobic Gram−/+ bacteria blocking the synthesis of peptidoglycan, an essential component of the bacterial cell wall by inhibiting the UDP-N-acetylglucosamine enolpyruvyl transferase, which catalyzes the first-step reaction for the synthesis of the bacterial cell wall [7,11]. Furthermore, an immunomodulatory effect has also been reported [7,12].

It is also worth noting that FOS exerts some action against biofilms, alone or in combination with other antibiotics.

Finally, toxicity is very uncommon such as gastrointestinal effects (e.g., nausea, vomiting and diarrhea), higher transaminases and local irritation at the intravenous injection site [13,14]. Regarding FOS distribution in target tissues, an optimal penetration in muscle, subcutaneous tissues, infected lungs, brain (particularly in meningeal inflammation) and a variable penetration in abscesses were demonstrated, depending on the vascular permeability and the perfusion of surrounding tissues [15,16].

Considering all the above-mentioned characteristics and their unique mechanisms of action, their uses are currently being revaluated for intravenous administration for the treatment of systemic infections by multidrug-resistant bacteria, attracting clinicians interest worldwide [17,18,19,20].

This re-evaluation is even due to the relative lack of new antibiotic molecules and to the higher incidence of multidrug-resistant microorganism infections [6].

Recently, some infrequent but successful uses were reported for the treatment of multidrug resistant pathogens [10,21,22,23].

Nowadays, generally acknowledged pharmacokinetic and pharmacodynamic (PK/PD) characteristics for FOS are lacking in the literature, and both concentration- and time-dependent activities have been suggested [18].

In detail, Roussos et al. [6] reported that the PK/PD profile of FOS may be organism-dependent. FOS exhibits concentration-dependent killing activity against *E. coli*, *P. mirabilis* and *Streptococcus pneumonie* and time-dependent bactericidal activity against *S. aureus* and *P. aeruginosa*. These PK/PD characteristics suggest possible benefits for the continuous infusion (CI) use for this drug.

Moreover, considering the significant risks of acquiring nosocomial infections, the high costs of hospitalization for the health systems and the will and/or the need to continue the treatment at home or in other structures, CI through elastomeric pumps can be extremely useful for therapeutic “bridging” strategies as well as for the outpatient parenteral antibiotic therapy (OPAT).

Focusing on OPAT through elastomeric pumps, one of the most critical factors for its feasibility is the active compound stability in the conditions of use [24]. In this context, this depends on the chemical/thermal stability of the compound and its interaction with the container surfaces at external body temperature.

Recent data suggest high thermal stability of FOS powders and solutions as well long stability, even at room temperature, in biological samples using different approaches ranging from Thermogravimetry (TG) and Differential Scanning Calorimetry (DSC) to liquid chromatography coupled with tandem mass spectrometry (LC-MS/MS) or microbiological testing [25,26,27,28,29,30,31].

It is important to mention that, considering the very low light absorption of FOS, the commonly applied techniques for stability testing such as LC-UV, PDA or Fluorimetry are not viable options.

Nevertheless, no evidence is currently available in the literature about FOS stability in elastomeric pumps. Therefore, this study aimed to evaluate that on the medium-term, both before administration (at 4 °C) and during administration at external body temperature (34 °C), according to the EMA and ICH guidelines for its use in the stability of medicinal products [32,33].

EUCAST guidelines for FOS intravenous use suggest a dosage between 12 g/die and 24 g/die, with 16 g/die as one of the most common dosages; therefore, the stability study was conducted at these doses, in the 250 mL volume of elastomeric pumps [34,35].

## 2. Materials and Methods

A stability study of InfectoFos^®^ 40 mg/mL (InfectoPharm s.r.l., Milan, Italy) preparation for intravenous use (consisting of 5.38 g of FOS disodium salt, corresponding to 4 g of FOS, per bottle) in elastomeric pumps was performed at 4 °C and 34 °C; 3 lots of FOS powder were analyzed. Samples were prepared at the concentration of 16 g in 250 mL and 24 g in 250 mL of water and of glucose for injectable preparations, collected in elastomers and stored at 4 °C and 34 °C, respectively, and analyzed on 9 consecutive days, following the EMA and ICH guidelines [32,33] for drug stability evaluation.

Microbiological testing was not performed during this study since it was focused on chemical stability. Hereafter, details about chemicals, preparation of the dilutions for the analysis via LC-MS/MS and the actual conditions/timings for the stability study are reported.

### 2.1. Chemicals

InfectoFos^®^ powder was kindly supplied by InfectoPharm s.r.l. FOS sodium salt analytical standard (purity 99%), Hexylamine (99%), Diethylamine (≥99.5%) and Acetic Acid (≥99%) were purchased from Sigma-Aldrich (Milan, Italy). Glucose (5%) solution for injectable preparations and water for injectable preparations were purchased from Eurospital (Trieste, Italy). Avibactam (AVI, >98%), used as internal standard (IS), was purchased from Alsachim (Illkirch Graffenstaden, France).

HPLC-grade acetonitrile and HPLC-grade methanol were obtained from VWR International (Radnor, PA, USA). HPLC-grade water was produced with Milli-DI system coupled with a Synergy 185 system by Millipore (Milan, Italy).

### 2.2. Preparation of Fosfomycin Solution

Regarding the stability study at 4 °C, 3 batches of InfectoFos^®^ powder were dissolved at a concentration of 16 g/250 mL in water for injections and put in a fridge, kept away from light up to 9 days. Similarly, for the experiment at 34 °C, 3 different batches of InfectoFos^®^ powder were dissolved at two concentrations of 16 and 24 g in 250 mL of glucose solution (5%) and put in a dry oven, away from light. The elastomeric pumps were loaded with a 50 mL syringe, using a female–female Luer-lock connector.

### 2.3. Stability at 4 °C and 34 °C

The preparation stability was evaluated for 5 consecutive days, plus further points at 9 days; each aliquot at each timing was compared to a freshly prepared solution.

Two calibration curves were prepared for each analysis: one from an analytical standard solution of FOS (99% purity) and the other one from the reconstitution of InfectoFos^®^ powder.

The calibration curves were analyzed in duplicate for each run, showing good linearity and mutual agreement. Before the analysis on each day, each elastomer injector was conditioned for 1 h, discarding the provided solution. Then, the injector was moved to a polypropylene tube to recover the solution for 1 h and vortex mixed for 10 s.

Samples were diluted 1:10,000 in triple replicates (*n* = 3) for each batch, at a final theoretical concentration of 6400 ng/mL (9600 ng/mL for the 24 g/250 mL) in vial and, for each replicate, injected in triplicate into the instrument (*n* = 3) for a total of nine repetitions (*n* = 9) for each lot and for each timing. These concentrations ensured linear signal, without saturation, during the method development and a simple/reliable preparation.

Regarding stability at 34 °C, it was evaluated up to 9 days, each aliquot at each timing was compared to a freshly prepared aliquot at the concentration indicated by the data sheet (5.28 g FOS disodium/100 mL, equivalent to 4 g/100 mL FOS) to ensure optimal solubility for comparison.

A double replicate calibration curve was prepared for each assay through reconstitution of InfectoFos^®^ powder, 5.28 g of disodium FOS, corresponding to 4 g of FOS, in 100 mL.

These curves showed optimal linearity (R^2^ > 0.996) at all timings analyzed.

For the preparation of the calibration curve, a 4 g/100 mL solution was 2500-fold diluted (final concentration 16 mg/L) and then diluted further 1:1 to obtain 4 calibrators, with concentrations of 2–4–8–16 mg/L.

A similar process was carried out for the preparations in the elastomers, which were diluted 10,000 folds, to fall within a measurable range. The dilution yielded to final concentrations in vials of 6.4 mg/L and 9.6 mg/L, respectively. In this case, before the analysis, the pump injector was appropriately conditioned (20 min drip time). The aliquot was then vortex mixed for at least 60 s. For each batch, concentration and timing, the samples were analyzed in triple replicate (*n* = 3).

### 2.4. Analytical Method

#### 2.4.1. Chromatographic Conditions

The chromatographic system used in this work was a LX50 UHPLC (Perkin Elmer), composed of an Integrity^®^ autosampler, a SPH1299^®^ Dual UHPLC Pump and a Mistral^®^ column oven. The chromatographic separation was achieved via ion-pairing chromatography using a Hypercarb column, 2.1 × 150 mm, 1.8 μm (Thermo Scientific, Milan, Italy) at 40 °C.

The sample manager’s temperature was set at 15 °C.

The flow rate was settled at 0.4 mL/min, with a gradient of two mobile phases: mobile phase A was 5 mM of Hexylamine, 0.4% Diethylamine and 0.2% Acetic Acid in HPLC-grade water, and mobile phase B was 5 mM of Hexylamine, 0.4% Diethylamine and 0.2% Acetic Acid in HPLC-grade acetonitrile.

In details, the chromatographic gradient started with 3% mobile phase B up to 1 min (for the analyte separation), then it was increased to 10% at 2 min and held at the same percentage up to 2.50 min (column washing); a linear decrease of 3% of mobile phase B was applied up to 2.60 min and held at the same percentage up to 6 min to the end of analysis, in order to achieve optimal re-equilibration. The total runtime was 8 min.

Water/methanol 95:5 *v*/*v* was used as weak washing solution, while water/acetonitrile 30:70 *v*/*v* was used as strong washing solution. Two strong washing and three weak washing cycles (250 µL each) were applied, sequentially, after each injection.

#### 2.4.2. Mass Conditions

Obtained samples were analyzed with a previously developed ultra-high liquid chromatography (UHPLC) coupled with tandem mass spectrometry method on a LX50^®^ UHPLC system equipped with a QSight 220^®^ (Perkin Elmer, Milan, Italy) tandem mass spectrometer.

Negative ionization was used, and the ionization efficiency and repeatability were controlled by evaluating the signal of a constant concentration (1000 ng/mL) of Avibactam, added to each vial immediately before the analysis, as an internal standard (IS).

“Zero-Air” (dry air) was used as nebulizing and heating gas, while nitrogen was used as drying and collision gas; both were produced at a high purity (>99.9%) with a Cinel Zefiro^®^ Combined (Cinel, Vigonza, Italy) gas generator. General mass parameters for negative ionization were electrospray voltage of −4.8 kV; source temperature of 300 °C; nebulizing gas flow of 300 L/h; heating gas flow of 350 L/h; drying gas flow of 130 L/h and Heated Surface Induced Desolvation (HSID) temperature of 300 °C. Optimization of the mass conditions has been performed via the infusion of reference standards of FOS and corresponding IS at the concentration of 1 ppm in water/methanol 50% *v*/*v* at 5.0 μL/min into the mass spectrometer, in combination with the flow from the chromatographic system at medium concentrations phases (Phase A and Phase B 50% *v*/*v*). FOS exhibited two transitions: 137.0 > 78.9 *m*/*z* and 137.0 > 62.8; the first was used to quantify (quantification trace), whereas the second was used to confirm peak identity (secondary ion trace), in accordance with previously reported methods [27,29,30,31,36,37,38]. Dwell Time was 25 ms, Entrance Voltage was −12 V and Collision Energy was 20 and 40 eV for the First Ion Trace and for the Second Ion Trace, respectively. Similarly, quantification and qualification traces for AVI (the IS) were 264.1 > 80.0 and 264.1 > 96.0, respectively.

#### 2.4.3. Method Validation and Stress Testing

Method validation following FDA and EMA guidelines was performed for the determination of quantification performance in terms of accuracy, precision, linearity, specificity and sensitivity. No evaluation of matrix effect or recovery was included since the injected included no biological matrix and consisted in dilutions of FOS disodium salt in pure water, without any sample preparation procedure other than simple dilution. A calibration curve with 4 levels (2, 4, 8 and 16 mg/L) and 2 quality control (QC 2.5 and 5 mg/L, respectively) samples were applied, considering the extremely narrow quantification range that was needed for the stability study. A linear fitting was applied, with 1/concentration weighing. The LLOQ was considered as the lowest point of the calibration curve (2 mg/L) since no investigation of lower concentrations was needed in this study. The method capability to monitor FOS degradation was tested through stress testing of FOS preparation at 4 conditions for 24 h: 4 °C (reference condition), 96 °C (only temperature challenge), 96 °C with pH of 2 (thermal + acid challenge with formic acid) and 96 °C with pH of 11 (thermal + basic challenge with ammonia). These preparations were analyzed after a 10,000-fold dilution.

## 3. Results

### 3.1. Method Validation and Stress Test Results

The LC separation yielded FOS and IS peaks at 1.64 and 2.6 min of retention time (RT), with a capacity factor (k′) of 1.5 and 3.1, respectively; this prevents the analytes to be eluted together with potentially unretained impurities present in the samples. No interfering peaks were observed at these timings during the analysis. Moreover, the RT remained stable throughout the whole stability study. All the calibration curves showed satisfactory linearity, with an r^2^ > 0.996. Accuracy and precision at quality control levels resulted >96.1% and in a CV% of 5.6%, respectively. Accuracy and precision (CV%) at the LLOQ (2 mg/L) were 102.2% and 6.2%, respectively. All the parameters fulfilled the requirements from the EMA and FDA guidelines for the validation of bioanalytical methods (accuracy within 85–115%, CV within 15% and mean r^2^ > 0.996). Through stress testing, the method was capable to highlight a significant degradation of FOS only in the acid condition (96 °C and pH 2), with a 28% degradation, while it resulted as stable (less than 2% difference) in the other conditions. The chromatogram of FOS after the stress testing is reported in Figure S3.

### 3.2. Pre-Administration Stability at 4 °C and In-Use/External Body Temperature Stability at 34 °C

**Figure 1:**
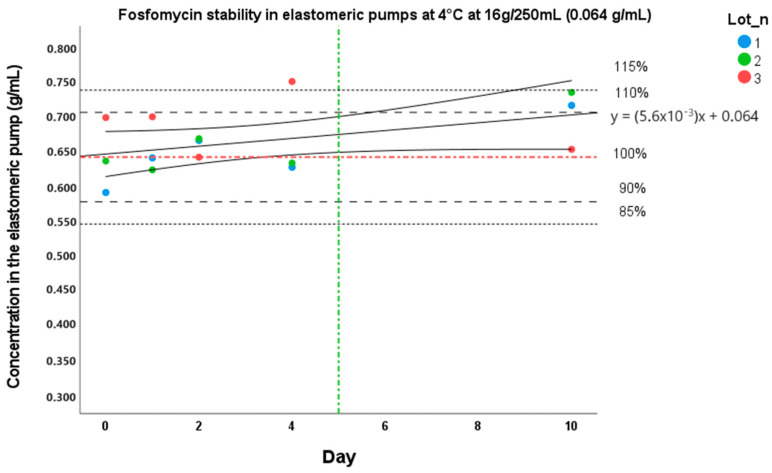
Stability of Fosfomycin at 16 g/250 mL at 4 °C in elastomer.

In this context, FOS resulted as stable in elastomeric pumps for 5 days from its preparation at a storage temperature of 4 °C, as shown in Figure 1. Results sorted by batch are summarized in Table 1. The obtained data are graphically shown as a percentage value compared to “time 0” (considered 100% stability). The results are indicated as the means of the three evaluations in the different lots. Since the results of the analysis session at the third day were inconsistent with the previous timings and with the following one (Supplementary Figures S1 and S2; this problem was related to a failure in the gas generator, which produced a variable flow of “zero air” to the MS source), an additional timing was carried out (day 10) in order to confirm the degradation trend of the preparation in Figure 1. It is important to note that the stability limit is settled, according to ICH guidelines [32,33], where a confidence interval boundary on the degradation curve meets the acceptability threshold (10% change). 

The continuous line represents the degradation curve, and the two curves represent the 95% interval of confidence. The red line stands for the nominal concentration value of the preparation (0.064 g/mL and 100% stability), while the upper and lower dashed lines represent the reference limit values (85% and 90–110–115%) of stability. The vertical green line represents the time limit of stability (5 days).

**Figure 2:**
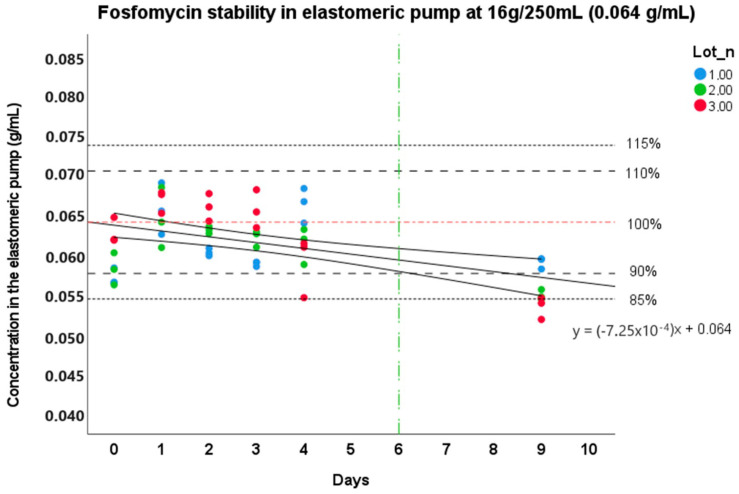
Stability of Fosfomycin at 16 g/250 mL at 34 °C in elastomer. The continuous line represents the degradation curve, and the two curves represent the 95% interval of confidence. The red line stands for the nominal concentration value of the preparation (0.064 g/mL and 100% stability), while the upper and lower dashed lines represent the reference limit values (85% and 90–110–115%) of stability. The vertical green line represents the time limit of stability (6 days).

**Figure 3:**
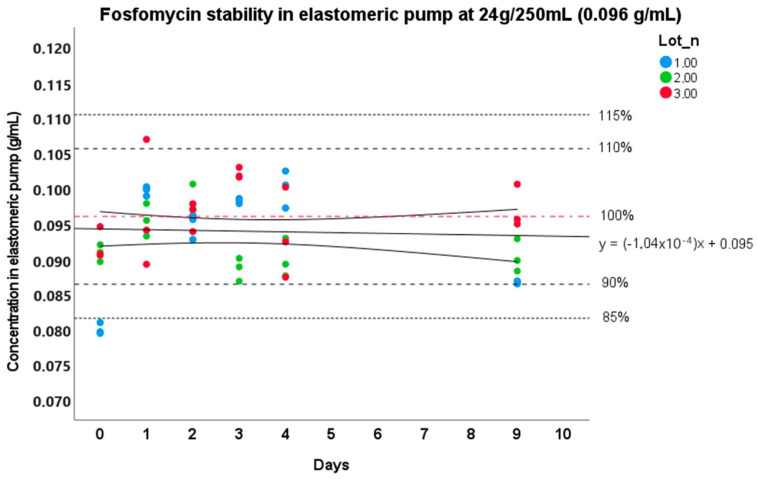
Stability of Fosfomycin at 24 g/250 mL at 34 °C in elastomer. The continuous line represents the degradation curve, and the two curves represent the 95% interval of confidence. The red line stands for the nominal concentration value of the preparation (0.096 g/mL and 100% stability), while the upper and lower dashed lines represent the reference limit values (85% and 90–110–115%) of stability.

Regarding stability at 34 °C, the trend curve with the confidence interval boundaries at 95% for the stability study at an external body temperature are reported in Figure 2 and Figure 3. As it can be seen in the figures, FOS resulted as stable up to 6 days in elastomeric pumps from its preparation at a storage temperature of 34 °C at the concentration of 0.064 g/mL, as highlighted by the lowest boundary of the 95% interval of confidence of the degradation curves. The highest concentrations showed a higher stability over time (even more than 9 days), exceeding the observation period. In detail, on the 5th day, the preparations showed a mean degradation of −2.9% (s.d. 5.8%) at the concentration of 16 g/250 mL and −1.6% (s.d. 5.3%) at 24 g/250 mL. On the 9th day, we observed −12.9% (s.d. 4.5%) and −4.9% (s.d. 5.5%) of degradation for the concentration of 16 g/250 mL and 24 g/250 mL, respectively.

## 4. Discussion

In this study, an ion pairing graphitic carbon reverse phase LC-MS/MS method was developed and validated, and it was applied to a stability study of FOS in elastomeric pumps, observing a satisfactory stability long enough to allow clinical use in the context of OPAT.

This can be crucial, since no previous study investigated medium-term pre-administration stability at 4 °C and in-use/external body temperature stability (34 °C) of FOS disodium salt for intravenous use (InfectoFos^®^, InfectoPharm s.r.l.) in elastomeric pumps; these stability data represent the basic condition to assess the eligibility of FOS for OPAT using CI in elastomeric pumps. The stability study was carried out at the Laboratory of Clinical Pharmacology and Pharmacogenetics, with certified PHASE I AIFA, UNI EN ISO 9001 and 13485 (CE-IVD) [39,40], following EMA stability guidelines.

In our study, an UHPLC-MS/MS method was developed and validated in order to evaluate the stability of the preparation. In fact, it was excluded to carry out the stability study in ultra-high performance liquid chromatography coupled to a photodiode array detector. This method was generally preferred for this type of analysis, due to the intrinsic characteristics of the molecule (that showed an absorbance only at non-specific wavelengths between 190–200 nm), which did not allow for reliable and reproducible analyses with this type of detector. Moreover, LC-MS/MS is associated with very high specificity, allowing the discrimination of FOS from any possible degradation product or impurity.

Some previous works described LC-MS/MS methods for the quantification of FOS in plasma or urine samples [27,29,30,31,36,37,38] and investigated FOS stability in these matrices as well in autosamplers after sample preparation, with encouraging results. The vast majority of these works used HILIC technology, with good analytical performance; nevertheless, considering that HILIC typically has a poor robustness on the medium-long term, we chose to use graphitic carbon chromatography with ion-pairing agents (diethylamine and hexylamine), obtaining enough retention performance, reliable RT and peak shape, as well as reproducible signal, using the same ESI transitions, which were used in previous works.

The whole study, both in the method validation phase and in the stability assessment, EMA/ICH guidelines were followed to increase the reliability of the obtained results and to standardize their reporting [32,33,41,42].

Outpatient parenteral antibiotic therapy (OPAT) administration offers several advantages: it guarantees an improvement in patient quality of life, allowing them to continue with their home daily routine despite receiving intravenous therapy. In addition, the potential risk of nosocomial infections can be reduced.

This therapy is also favorable from an economic perspective for the healthcare systems because it reduces the mean number of hospitalization days [43,44]. Considering the portable elastomeric infusion pumps, they are focused on making the administration easier for patients; they are functional devices for the delivery of intravenous therapies [45].

The elastomeric reservoir, commonly made of polyisoprene, latex or silicone, which contains the medication, is stored in a transparent plastic device and is able to release the drug solution at a constant flow along an infusion line [46].

These pumps have several advantages, providing great autonomy to the patient: safety (it is a perfectly sealed container, which guarantees sterility if it was filled in a sterile environment), portability (about 300 mL of volume and 300 g of weight), non-electronic manipulation, continuous drug administration at a constant flow rate, silence and simple management by the patient or caregiver. All these characteristics provide great benefit to the patient, both from a PK/PD point of view, increasing the overall exposure—T > MIC—with less intra-day variability in the concentrations, and by a safety point of view for the patient, reducing the risk of nosocomial infections and the emergence of antimicrobial resistance.

In addition, as mentioned above, they have a better cost effectiveness profile compared to the electronic ones [47].

On the other hand, some drawbacks are also present: the lack of an alarm, if a failure happens during the drug delivery, unlike electronic devices; lower delivery rate accuracy than electronic pumps and the necessity to choose a specific speed through the whole administration. Moreover, environmental factors, such as external temperature, that could affect the stability of antibiotics, need to be considered [8,9].

Considering all these aspects, antimicrobial stability is an essential point to guarantee an optimal health outcome [10]. In fact, instability of the active compound could lead to underdosing, with the risk of therapeutic failure and emergence of antimicrobial resistance as well as possible toxic effects from degradation products.

Many aspects could affect drug stability, including amount of the drug; the use of additives and diluents; storage parameters, such as the temperature collection and time, and the composition of the used elastomeric pump. Consequently, stability data are very important requirements to guarantee safety management; particularly, thermal stability is crucial for elastomeric pumps, since these are often at close contact with the patient’s skin, therefore at an external body temperature (about 34 °C) for a medium–long period.

In the literature, stability in elastomers has been widely investigated for several antibiotics, with respect to the results published regarding antifungals and antivirals [48,49,50].

β-lactams (in particular, penicillin and cephalosporins) are the groups of antibiotics that are more studied; stability results regarding antimicrobials are concordant, with some exceptions. As an example, for ceftazidime, results reported in the literature differs, but the instability of this antibiotic seems to occur at a high concentration; on the other hand, poor stability of flucloxacillin in elastomers is associated with high temperatures [51].

FOS showed sufficient stability at the analyzed concentrations, confirming its theoretical technical suitability to be used for OPAT.

In detail, the InfectoFos^®^ FOS in elastomer is stable for 5 days from its preparation at a storage temperature of 4 °C, since a slight trend in increasing concentrations of FOS solutions during this study was observed. It can be explained by a possible formation of micro-precipitates and slight lack in homogeneity of the preparation in the initial volumes near to the connector, which can theoretically be favored at a low-temperature condition. Additionally, day 0 concentrations were variable between lots and were lower than expected (mean of 5800 ng/mL observed vs. the 6400 ng/mL expected) suggesting that priming time should be longer at 4 °C. This phenomenon could be attributed to a sub-optimal mixing in the first hours, inside the elastomeric pump or in the tubes, or to a slight adsorption phenomenon to the tubes (this evaluation was obtained through a comparison with the standard produced and diluted in glass containers; the data were not reported).

Regarding real life temperatures, FOS stability tests were performed at 34 °C, as reported in other studies in the literature as the external body temperature [48,49]. FOS resulted as stable for at least 6 days after its preparation at the concentration of 16 g/250 mL. Regarding the preparation at 24 g/250 mL, the limit of stability cannot be precisely determined, but it can be certified that it exceeds the limit of 9 days set for this study. Additionally, the extent of the suitability of FOS for CI in the outpatient context should be further investigated by assessing PK values in real patient samples and evaluating the clinical outcome. This evidence of very high stability is in accordance with previous data about thermal stability [25,26] as well as with our results from stress testing, where no significant degradation was observed at 96 °C for 24 h, neither in neutral nor in basic conditions.

The choice to omit microbiologic testing was due to the focus on chemical stability as well as the fact the expected and very high chemical stability underlines also retained antimicrobial effect and the fact that LC-MS/MS was applied. In fact, this technique is extremely specific, ensuring the identity of the monitored peak, so that no degradation product or altered molecule could be mistaken for the microbiologically active compound, potentially leading to mismatches between LC-derived stability data and microbiologic testing.

A limitation of this study is that it is focused on a single type (polyisoprene, which is actually one of the most popular) of material; therefore, no inference about stability in other materials (latex or silicone) can be made. Anyway, polyisoprene offers several advantages over other materials, such as acid-base, alcohol and polar solvent resistance, and it is compatible with low-temperature storage and, compared to latex, is safe for atopic individuals. Moreover, further investigation would be beneficial for higher dosages, covering more days of treatment or even a full treatment course. These investigations will be explored in future stability studies.

## 5. Conclusions

In conclusion, the information provided in this work should be considered in the treatment of patients within the Outpatient Parenteral Antibiotic Therapy setting.

This is the first study assessing the technical feasibility of using FOS for outpatient parenteral antibiotic therapy, up to at least 5 days; in fact, its stability at 34 °C in elastomeric pumps makes this drug a possible good candidate to be used for continuous infusion in difficult-to-treat infections and as a therapeutic “bridging” strategy.

Further stability studies are needed to confirm the appropriate use of the FOS including the relationship between the material of the elastomeric pump and drug stability.

Therefore, this approach should be tested soon in a dedicated clinical and pharmacokinetic study, in order to evaluate the possible gains both in the pharmacokinetic profile and in the clinical effectiveness as well as the likely miscibility and stability with other antibiotics in the context of an association therapy.

## Figures and Tables

**Table 1:** Stability of three lots of Fosfomycin (InfectoFos^®^) concentrations in an elastomeric infusion pump for 10 days, except day 3.

	Day 0		Day 1		Day 2		Day 4		Day 10	
	CV%	Stability (%)	CV%	Stability (%)	CV%	Stability (%)	CV%	Stability (%)	CV%	Stability (%)
**Batch 1**	5.8	100	12.5	108	1.3	113	15.9	106	9.3	121
**Batch 2**	5.7	100	10.8	98	1.9	105	16.3	100	5.9	115
**Batch 3**	6.1	100	8.0	100	13.5	92	13.2	107	9.1	93

## Data Availability

Data will be provided on request.

## Supplementary Materials

The following supporting information can be downloaded at https://www.mdpi.com/article/10.3390/pharmaceutics15092347/s1, Figure S1: Three lots of Fosfomycin (InfectoFos^®^) concentrations in an elastomeric infusion pump stored for 5 days at 4 °C, Figure S2: Three lots of Fosfomycin (InfectoFos^®^) concentrations in an elastomeric infusion pump stored for 5 days at 4 °C, except day 3; Figure S3: Stress test evaluation after 24 °C at 4 different conditions. As can be observed, FOS resulted stable at 96 °C in neutral and basic conditions, while it showed 28% degradation at pH 2 (96 °C).
