# Supplementary material for: Stability Study of Fosfomycin in Elastomeric Pumps at 4 °C and 34 °C: Technical Bases for a Continuous Infusion Use for Outpatient Parenteral Antibiotic Therapy
Source: Pharmaceutics. 2023 Sep 19;15(9):2347. doi: 10.3390/pharmaceutics15092347 (PMC10537177; doi:10.3390/pharmaceutics15092347)
Supplement: Supplementary file 1 [file pharmaceutics-15-02347-s001.zip › pharmaceutics-2561844-supplementary.pdf]

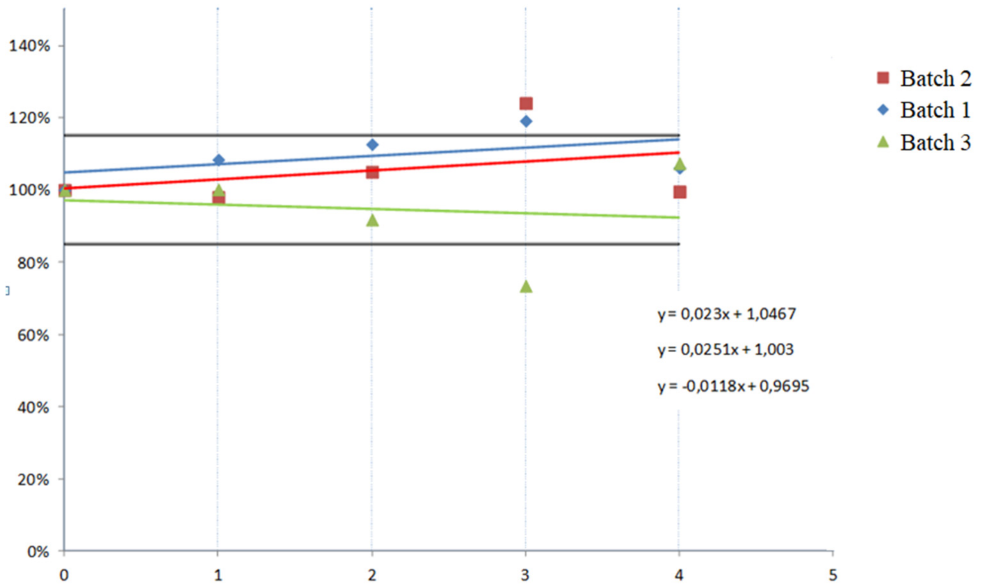

Supplementary Figure S1. Three lots of Fosfomycin (InfectoFos®) concentrations in an elastomeric infusion pump stored for 5 days at 4°C.

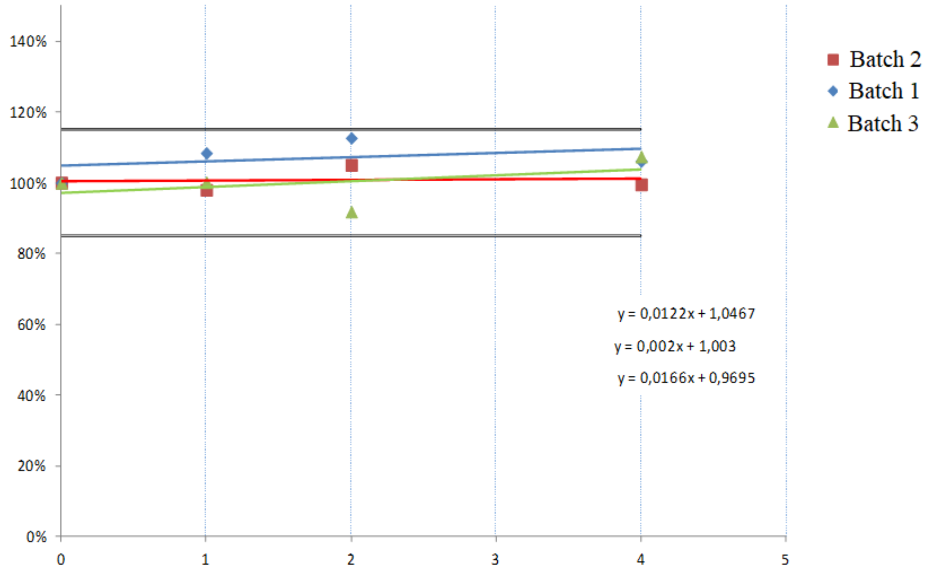

Supplementary Figure S2. Three lots of Fosfomycin (InfectoFos®) concentrations in an elastomeric infusion pump stored for 5 days at 4°C, except day 3.

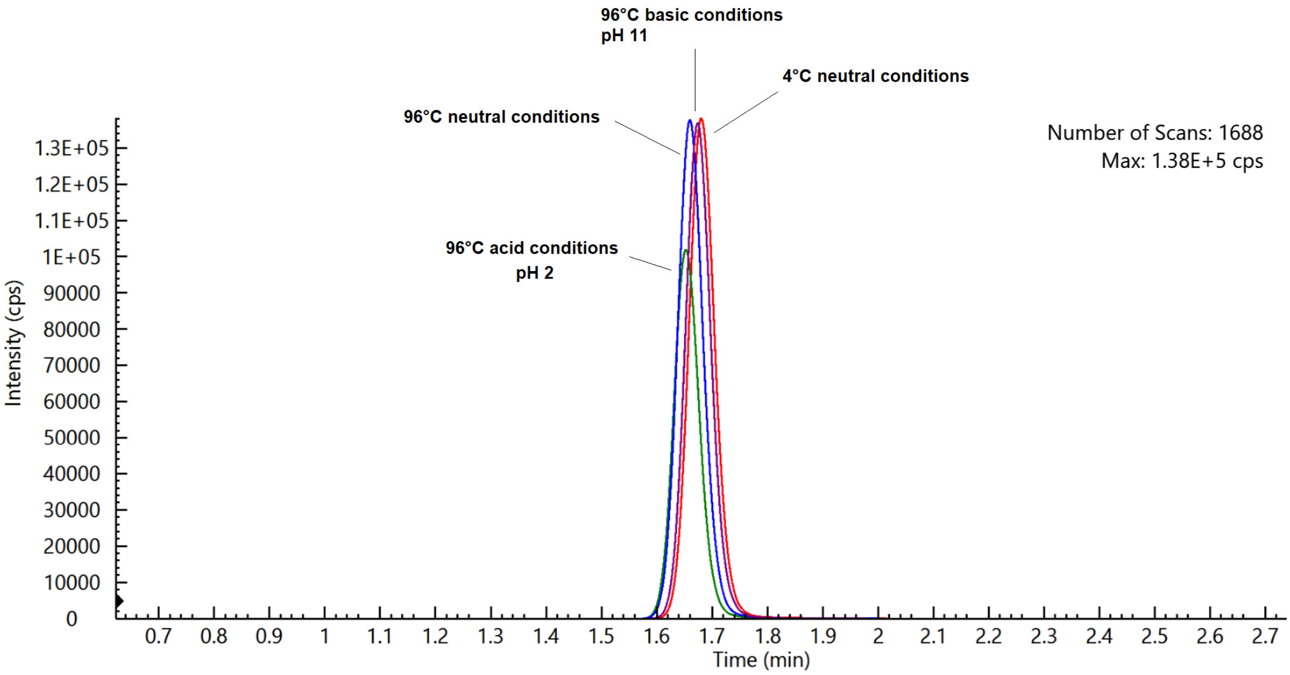

Supplementary Figure S3. Stress test evaluation after 24°C at 4 different conditions. As can be observed, FOS resulted stable at 96°C in neutral and basic conditions, while it showed 28% degradation at pH 2 (96°C).
